# The Role of Circulating Serotonin in the Development of Chronic Obstructive Pulmonary Disease

**DOI:** 10.1371/journal.pone.0031617

**Published:** 2012-02-03

**Authors:** Way K. W. Lau, Moira M. W. Chan-Yeung, Benjamin H. K. Yip, Amy H. K. Cheung, Mary S. M. Ip, Judith C. W. Mak, and the COPD Study Group of the Hong Kong Thoracic Society

**Affiliations:** 1 Division of Respiratory Medicine, Department of Medicine, The University of Hong Kong, Hong Kong SAR, China; 2 Department of Psychiatry, The University of Hong Kong, Hong Kong SAR, China; 3 Department of Pharmacology and Pharmacy, The University of Hong Kong, Hong Kong SAR, China; Helmholtz Zentrum München/Ludwig-Maximilians-University Munich, Germany

## Abstract

**Background:**

Cigarette smoking is a major risk factor in the development of age-related chronic obstructive pulmonary disease (COPD). The serotonin transporter (SERT) gene polymorphism has been reported to be associated with COPD, and the degree of cigarette smoking has been shown to be a significant mediator in this relationship. The interrelation between circulating serotonin (5-hydroxytyptamine, 5-HT), cigarette smoking and COPD is however largely unknown. The current study aimed at investigating the mediation effects of plasma 5-HT on cigarette smoking-induced COPD and the relation between plasma 5-HT levels and age.

**Methods:**

The association between plasma 5-HT, age and COPD was analyzed in a total of 62 COPD patients (ever-smokers) and 117 control subjects (healthy non-smokers and ever-smokers). Plasma 5-HT levels were measured by enzyme-linked immuno assay (EIA).

**Results:**

The elevated plasma 5-HT levels were significantly associated with increased odds for COPD (OR = 1.221, 95% CI = 1.123 to 1.319, *p*<0.0001). The effect remained significant after being adjusted for age and pack-years smoked (OR = 1.271, 95% CI = 1.134 to 1.408, *p* = 0.0003). Furthermore, plasma 5-HT was found to mediate the relation between pack-years smoked and COPD. A positive correlation (r = 0.303, *p* = 0.017) was found between plasma 5-HT levels and age in COPD, but not in the control subjects (r = −0.149, *p* = 0.108).

**Conclusion:**

Our results suggest that cigarette smoke-induced COPD is partially mediated by the plasma levels of 5-HT, and that these become elevated with increased age in COPD. The elevated plasma 5-HT levels in COPD might contribute to the pathogenesis of this disease.

## Introduction

Chronic obstructive pulmonary disease (COPD) is characterized by a progressive, nearly irreversible airflow obstruction and an abnormal inflammatory response in the lung [1]. The pathological changes of COPD include emphysema and/or bronchitis [2]. Diagnosis of COPD is based on spirometry which is measured by the ratio of force expiratory volume in 1 second (FEV_1_) to force volume capacity (FVC; FEV_1_/FVC) [3]. FEV_1_ alone reflects the severity of the disease [4]. COPD is predicted to become the third leading cause of death by 2020 [5], and yet there is no effective treatment. The risk of developing COPD grows with age, while the aging population is one of the major concerns around the world. According to the U.S. Census Bureau international database released in 2002, the proportion of the population aged 65 and above will constitute more than 20% of the total population in developed countries by 2025 [6]. Therefore, it is important to investigate possible predictors in order to prevent the disease at an early stage. One Hong Kong-based study has shown that smoking is an important predictor of COPD [7]. However, exploring other factors is necessary in order to gain a better understanding of the etiology of the disease.

Serotonin (5-hydroxytyptamine, 5-HT) is a neurotransmitter that plays an important role in pulmonary function. Reduction of plasma 5-HT levels has been associated with age in an *in vivo* study [8]. Current studies on the association between 5-HT and COPD focus mainly on the polymorphism of the serotonin transporter gene and pulmonary hypertension [9]. The serotonin transporter (SERT) is a membrane bound protein that controls the transport of 5-HT and has been shown to regulate plasma 5-HT levels [10]. The severity of pulmonary hypertension was positively associated with the frequency of the L-allele of the SERT gene in COPD [11]. A genome-wide association study (GWAS) based on more than 20,000 Europeans has demonstrated five loci that are significantly associated with lung function, including 5-HT receptor 4 (5-HTR_4_) [12]. More recently, Ishii and colleagues suggest that the degree of cigarette smoking may partially mediate the relation between SERT gene (SLC6A4) variation and COPD pathogenesis [13]. However, the interrelation between smoking, circulating 5-HT levels and COPD is still unclear.

On the basis of these previous findings, we hypothesized that plasma 5-HT contributes to the development of cigarette smoke-induced COPD. The present study aimed at investigating the mediation effects of plasma 5-HT levels on the relation between pack-years smoked and COPD, and at studying the correlation between plasma 5-HT levels and age in the COPD patients as well as in the control subjects.

## Materials and Methods

### Ethics statement

This study was approved by the Ethics Committee of Institutional Review Board of the University of Hong Kong/Hospital Authority Hong Kong West Cluster (HKU/HA HKW IRB, UW 04-058 T/380) and all participants provided written informed consent.

### Study design

This study followed the STROBE-guidelines (strengthen the reporting of observational studies in epidemiology) [14]. Pulmonary function parameters were measured according to the American Thoracic Society guidelines [15]. The pre-bronchodilator FEV_1_ and FVC values (% predicted) were used in this study. The reference values were based on our local population [16]. All COPD patients showed limited (<10% FEV_1_ % predicted) reversibility after the application of the bronchodilator. Information such as smoking habits, pack-years smoked, respiratory symptoms and other diseases were obtained by questionnaire. Subjects with a history of asthma, other airway diseases or ischemic heart disease were excluded.

### Participants

One hundred and seventy-nine male subjects were randomly selected from the COPD database conducted by the COPD Study Group of the Hong Kong Thoracic Society between 2005 and 2006 [7]. Stable COPD patients were recruited from out-patient respiratory clinics and defined according to the COPD guideline published in the Global Initiative for Chronic Obstructive Lung Disease (GOLD) [4], with FEV_1_/FVC<70 and/or FEV_1_<80 (% predicted) and no exacerbation in the past 12 weeks prior to the recruitment. They were either current smokers or ex-smokers (defined as those who have not smoked for at least one year). Control subjects were recruited from churches and community centers for the elderly in Hong Kong. They were subdivided into healthy non-smokers and ever-smokers, including current and ex-smokers. They were defined as control subjects by a measurement of FEV_1_/FVC≥70 and FEV_1_≥80 (% predicted) and had no chronic respiratory symptoms.

### Preparation of platelet poor plasma and 5-HT measurement

The venous blood samples obtained from all subjects were centrifuged at 1600×g at 4°C for 10 minutes. The platelet poor plasma was carefully collected and stored at −80°C until further analysis. Plasma 5-HT levels were measured using commercially available enzyme-linked enzyme-linked immuno assay (EIA) kits (Enzo Life Sciences, Plymouth Meeting, USA). The detection range of the kit was 0.49–500 ng/ml.

### Statistical analysis

Values are expressed as mean ± SD for normally distributed variables and median (interquartile range [IQR]) for non-normally distributed variables. Normality was tested by the Kolmogorov-Smirnov test. Demographic data and 5-HT levels between the two groups were compared by the two-tailed independent Student's t-test, the Mann-Whitney U test or the χ^2^ statistics, where appropriate. The regression and mediation analyses were performed within healthy ever-smokers and COPD patients, since all of our COPD patients are ever-smokers.

Binary logistic regression analysis was applied to estimate the strength of association between the risk of developing COPD and plasma 5-HT levels, odds ratios (OR) with 95% confidence intervals (CI) were calculated (before and) after adjusting for age and pack-years smoked.

Mediation analysis with dichotomous outcomes was implemented to identify the potential mediating effect of plasma 5-HT levels on the relation between pack-years smoked and COPD, based on the procedures described by Herr [17]. Briefly, the total effect of pack-years smoked (i.e. initial variable) on COPD occurrence (i.e. outcome) was expressed as the sum of their direct and indirect effects. The indirect effect was obtained from the product of the effect of pack-years smoked (i.e. initial variable) on plasma 5-HT levels (i.e. mediator) and the effect of plasma 5-HT levels on COPD occurrence (i.e. the outcome), while controlling for the pack-years smoked. The total, direct and indirect effects were obtained from the coefficients of different regression models. Since plasma 5-HT levels were not normally distributed, a log-transformation of this variable was performed in the linear regression model. The significance of the indirect mediation effect was determined using the Sobel test [18]. All the regression analyses performed for computing the regression coefficients for the mediation analysis were adjusted for age. Missing values were handled by excluding cases list-wise for all regression analyses.

Spearman's correlation analysis was applied to study the relation between age and plasma 5-HT levels. The Statistical Package for the Social Sciences (SPSS/PASW, version 18.0, Chicago, IL, USA) was used for statistical analyses. A *p*-value of less than 0.05 was regarded statistically significant.

## Results

### Subject demographics

The demographic characteristics of all studied subjects were summarized in Table 1. Stable COPD patients were significantly older than healthy non-smokers or ever-smokers. The number of pack-years smoked was significantly higher in the COPD patients than in healthy ever-smokers. There were missing data with respect to the pack-years smoked (10.8%) in healthy ever-smokers due to incomplete information in the questionnaires. The COPD patients had significantly lower lung function (FEV_1_ % predicted, FVC % predicted and FEV_1_/FVC ratio) compared to the control subjects.

**Table 1:** Characteristics of study subjects.

	Healthy non-smokers	Healthy ever-smokers	COPD ever-smokers
Sample size	52	65	62
Age	57.6±19.5	64.4±15.0	b c72.8±6.9
Smoking status (Current/ex-smoker)	—	22/43	22/40
Pack-years smoked (Missing value %)	—	24.9±17.4 (10.8)	c54.7±37.1
FEV_1_, % predicted	102.4±12.0	101.2±11.2	b c37.8±12.8
FVC, % predicted	100.5±10.8	100.7±10.5	b c66.5±18.5
FEV_1_/FVC	79.2±5.8	a76.3±5.4	b c41.6±9.1

Values are mean ± SD.

FEV_1_ = force expiratory volume in one second, FVC = force vital capacity.

a *p*<0.01 compared to healthy non-smokers.

b *p*<0.001 compared to healthy non-smokers.

c *p*<0.001 compared to healthy ever-smokers.

### Plasma levels of 5-HT

**Figure 1:**
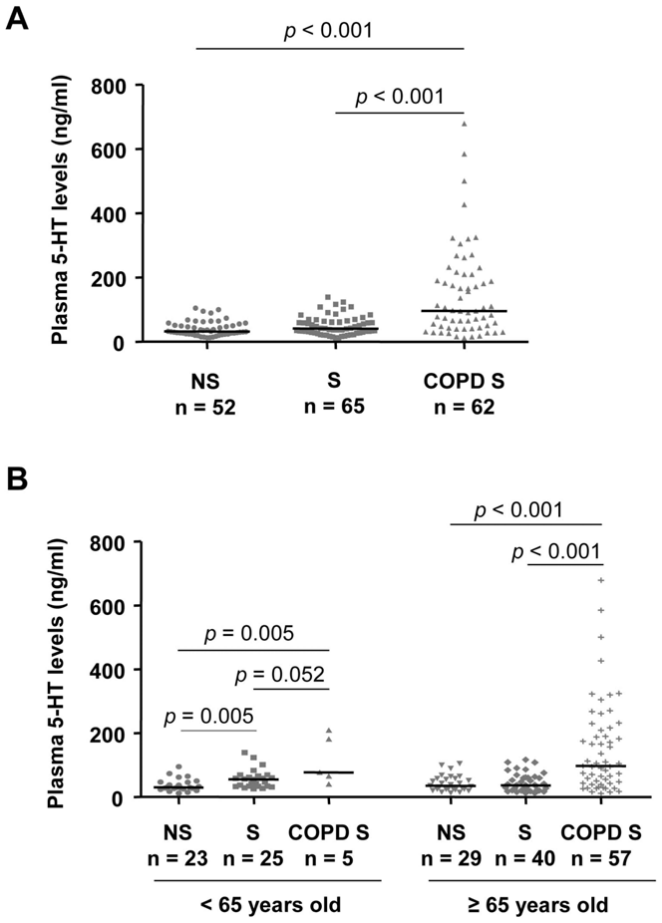
Plasma 5-HT levels across COPD patients and control subjects. (A) Plasma 5-HT levels of healthy non-smokers (NS), healthy ever-smokers (S) and COPD ever-smokers (COPD S). (B) Plasma 5-HT levels of healthy non-smokers (NS), healthy ever-smokers (S) and COPD ever-smokers (COPD S) in those <65 years old and ≥65 years old. Solid horizontal line represents the median value. *P* values were obtained from the Mann-Whitney U test.

**Figure 2:**
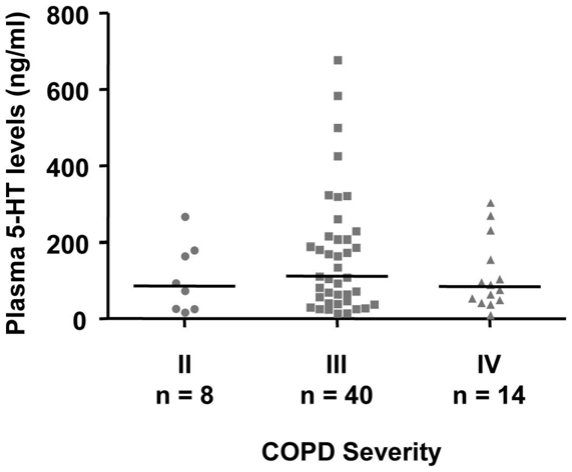
Plasma 5-HT levels of COPD patients with different stages. Solid horizontal line represents the median value. No significant difference was found across the groups.

**Figure 3:**
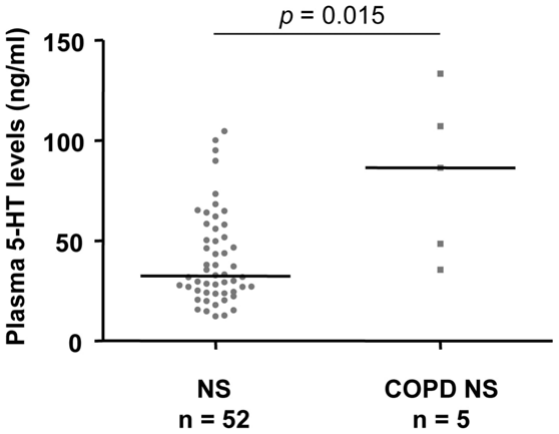
Preliminary findings on the plasma 5-HT levels of COPD non-smokers and control subjects. Plasma 5-HT levels of healthy non-smokers (NS) and COPD non-smokers (COPD NS). Solid horizontal line represents the median value. *P* values were obtained from the Mann-Whitney U test.

The plasma 5-HT levels were significantly higher in the COPD patients (96.6 ng/ml [43.6–209.8]; median [IQR]) than in either healthy non-smokers (32.4 ng/ml [24.2–54.9]) or ever-smokers (41.4 ng/ml [28.5–62.9], Figure 1A). We subdivided the three study groups into two subgroups of participants younger than 65 and those aged 65 or older, using the 65 year-mark as the cut-point. In those younger than 65 years of age, we found that plasma 5-HT levels were significantly higher in healthy ever-smokers (n = 25, 55.5 ng/ml [33.7–67.7]) than in healthy non-smokers (n = 23, 30.1 ng/ml [25.2–46.7]). The plasma 5-HT levels of COPD ever-smokers in this subgroup (n = 5, 77.7 ng/ml [53.1–195.8]) were significantly higher than those of healthy non-smokers, and higher than those of healthy ever-smokers at borderline level. In those 65 or over, the plasma 5-HT levels were significantly higher in COPD ever-smokers (n = 57, 97.6 ng/ml [43.4–213.6]) than in either healthy non-smokers (n = 29, 35.5 ng/ml [23.9–58.3]) or healthy ever-smokers (n = 40, 37.2 ng/ml [21.7–61.1], Figure 1B). There were no significant differences in plasma 5-HT levels across COPD severity (Figure 2). In another subgroup analysis with limited sample size, our preliminary data showed that the plasma 5-HT levels of COPD non-smokers (n = 5, 86.5 ng/ml [42.0–120.4] were also significantly higher than those of healthy non-smokers (Figure 3).

Since 5-HT levels are reported to correlate negatively with blood pressure [19], we examined the plasma 5-HT levels in COPD patients with or without hypertension. There were 15 COPD patients with hypertension in this cohort. In the subgroup analysis, there was no significant difference (*p* = 0.96) in the plasma levels of 5-HT between COPD patients with (106.2 ng/ml [65.5–181.7]) and those without hypertension (5-HT: 94.8 ng/ml [43.0–217.3], data not shown). Therefore, we included subjects with hypertension in all the analyses in this study.

### Association between plasma 5-HT levels and COPD

The risk of developing COPD was analyzed by including plasma 5-HT levels, age, and pack-years smoked in the binary logistic regression model. The odds ratios were calculated according to a 10-units change of 5-HT levels. Plasma 5-HT levels were significantly associated with an increased odds for COPD (OR = 1.221, 95% CI = 1.123 to 1.319, unadjusted). This association remained significant after adjusting for age and pack-years smoked (OR = 1.271, 95% CI = 1.134 to 1.408, Table 2).

**Table 2:** Association between plasma levels of 5-HT and COPD in ever smokers with or without COPD by binary logistic regression analysis.

			a95% CI		
	aOR	aSE	Lower bound	Upper bound	b *P* value
5-HT (Unadjusted)	1.221	0.098	1.123	1.319	**<0.0001**
5-HT (cAdjusted)	1.271	0.137	1.134	1.408	**0.0003**

OR = odds ratio, SE = standard error, CI = confidence interval.

a Corresponding to a 10-unit change of the plasma 5-HT level.

b According to Wald test.

c Adjusted for age and pack-years smoked.

### Mediation effects of 5-HT on the relation between pack-years smoked and COPD

We found a significant association between pack-years smoked and COPD. Pack-years smoked were also significantly associated with plasma 5-HT levels. The mediation effect of plasma 5-HT levels on the relation between pack-years smoked and COPD was found to be significant (*p* = 0.035) using the Sobel test. Also, the direct effect of pack-years smoked on COPD was significant (*p* = 0.001) in the presence of plasma 5-HT, indicating a partial mediation effect of plasma 5-HT levels on the degree of smoking and the development of COPD (Table 3).

**Table 3:** Mediation effects of 5-HT on the relation between pack-years smoked and COPD.

	Effect	Estimate	SE	*P* value
Initial: Pack-years smoked	aTotal: Pack-years smoked on COPD	0.050	0.011	**0.000**
Mediator: Serotonin (5-HT)	bPack-years smoked on 5-HT	0.008	0.003	**0.003**
Outcome: COPD	c5-HT on COPD given pack-years smoked	0.024	0.007	**0.000**
	dIndirect: Pack-years smoked on COPD through 5-HT	0.483	0.046	**0.035**
	eDirect: Pack-years smoked on COPD given 5-HT	0.046	0.014	**0.001**

Since the level of 5-HT was not normally distributed, a log-transformation was performed in the linear regression model. All analyses were adjusted for age.

SE = standard error.

a,b,c,e Regression analyses were performed to calculate parameter estimates.

d The estimate is the product of the estimates of b and c. After the estimate and SE of the product were calculated, the Sobel test was performed to assess the significance of the indirect effect.

### Correlation between plasma 5-HT levels and age

**Figure 4:**
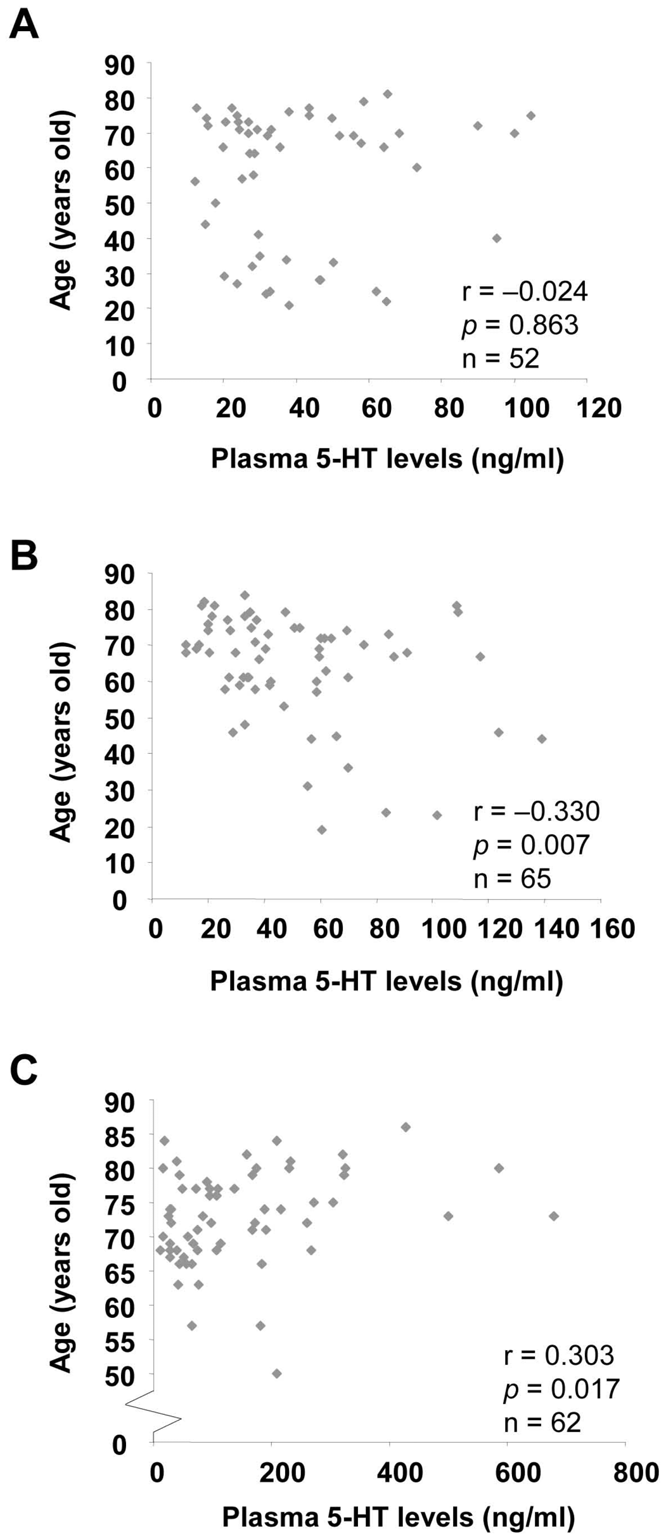
Spearman's correlation between plasma 5-HT levels and age in different groups. The scatter plot of the relation between plasma 5-HT levels and age in healthy non-smokers (A), healthy ever-smokers (B) and COPD ever-smokers (C). The Spearman correlation coefficient (r) and the *p* value were shown.

In the control subjects (healthy non-smokers and ever-smokers), we found no significant correlation between plasma 5-HT levels and age (r = −0.149, *p* = 0.108). In the COPD patients however, plasma 5-HT levels were positively associated with age (r = 0.303, *p* = 0.017). In the subgroup analysis, there was no association between plasma 5-HT levels and age in healthy non-smokers (r = −0.024, *p* = 0.863), but a negative association in healthy ever-smokers (r = −0.330, *p* = 0.007) (Figure 4). Since the COPD patients are significantly older than the control subjects, we tried to match the age between control subjects and COPD patients with ≥65 years of age and performed the correlation analysis again. There was still no correlation between plasma 5-HT and age in the control subjects (healthy non-smokers and ever-smokers) (r = −0.128, *p* = 0.295, n = 69), but a significant positive correlation in the COPD patients (r = 0.344, *p* = 0.009, n = 57) (data not shown).

## Discussion

This is the first report to demonstrate the association between plasma 5-HT levels, age, and COPD. Higher plasma 5-HT levels were found in stable COPD patients compared to the control subjects, which did, however, not correlate with the severity of the disease. Plasma 5-HT was a significant mediator of the relation between pack-years smoked and COPD. We also demonstrated a positive correlation between plasma 5-HT levels and age in the COPD patients, but not in the control subjects.

Platelet activation is one of the mechanisms that induce the release of 5-HT into the circulating blood. Nicotine, a major component in cigarette smoke, has been reported to stimulate the release of 5-HT *via* platelet activation *in vitro* [20]. In agreement with the literature, we found significant higher plasma 5-HT levels in healthy ever-smokers compared to those of healthy non-smokers with <65 years of age. Such difference disappeared in those ≥65 years of age, which might be due to the lowering effect of plasma 5-HT levels in ever-smokers with an increase in age. One possible mechanism for the elevated plasma 5-HT levels in COPD patients is the persistent platelet activation found in patients with stable or acute exacerbation of COPD [21]. Increased plasma 5-HT levels might be taken up by platelets *via* SERT. That way, SERT would be modified and downregulated due to the increased intracellular 5-HT levels in platelets, resulting in the inhibition of 5-HT reuptake [22]. These processes might further increase the plasma 5-HT levels in COPD.

**Figure 5:**
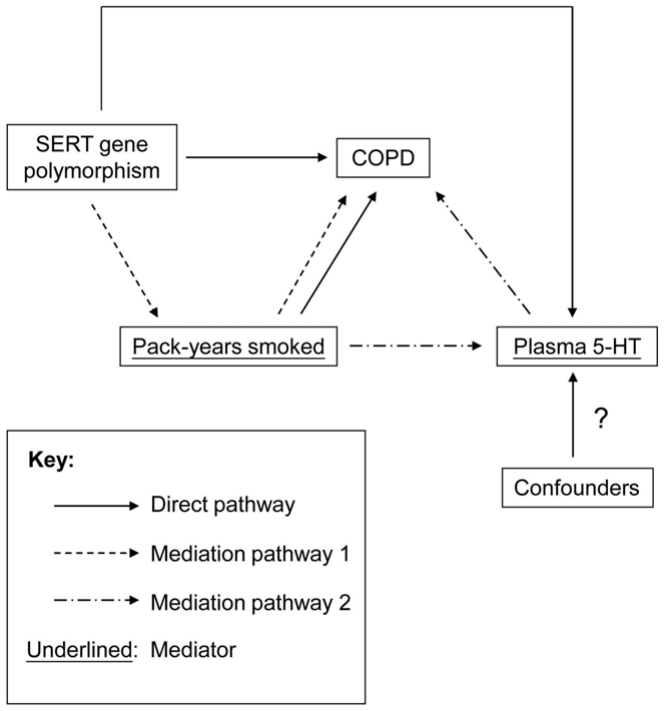
Hypothetic diagram for the relation among SERT gene polymorphism, plasma 5-HT, pack-years smoked and COPD. Pack-years smoked exert both direct and mediation effects on COPD.

Plasma 5-HT levels have been shown to be correlated with clinical severity and pulmonary function in symptomatic asthma patients [23]. In contrast, we found no association between plasma 5-HT levels and disease severity in COPD patients. This discrepancy may be due to the fact that the pathogenesis between asthma and COPD is different [24]. Another possible reason is that all the COPD patients in this study are stable. Here, we reported the partial mediation effect of plasma 5-HT on the relation between pack-years smoked and COPD. Ishii and colleagues have shown that cigarette smoking is a significant mediator of the relation between SERT polymorphism and COPD [13]. Whether the increased plasma 5-HT levels in COPD patients are due to SERT gene polymorphism remains unclear. In a small group of male COPD non-smokers, significant higher plasma 5-HT levels were observed in comparison to healthy non-smokers, suggesting that the plasma 5-HT levels are influenced by other factors apart from cigarette smoking (Figure 5). For example, COPD comorbidities such as increased pulmonary artery pressure or vascular dementia might be associated with the high plasma 5-HT levels [25]–[26]. Further studies are required to shed more light on these contributing factors and their possible role in altering plasma 5-HT levels in patients with COPD.

The relation between 5-HT levels and age was investigated in a study using different components of blood [27], demonstrating no correlation between plasma 5-HT levels and age, regardless of gender and smoking status. These findings are in agreement with the results obtained from our control subjects (healthy non-smokers and ever-smokers). However, their subjects were much younger (36.0±11.5) than our control subjects, and the effect of gender on the association between 5-HT and age was not addressed in the study. Another study reports a negative correlation between plasma 5-HT levels and age in women with age ranging from 40–84 years [28], regardless of smoking status, similar to male healthy ever-smokers in this study. Since there was no significant difference in age between healthy non-smokers and ever-smokers in our samples, our data suggest that healthy ever-smokers may be able to maintain the homeostasis of circulating 5-HT by lowering its level with an increase in age. In contrast, we found a positive correlation between plasma 5-HT levels and age in COPD ever-smokers. These data suggest that the regulation of plasma 5-HT levels is disturbed in COPD patients. A disturbance in any of the regulatory processes of 5-HT, including uptake, storage, release and breakdown by monoamine oxidases (MAOs) in the aging process or during the development of COPD can result in changes in the plasma levels of 5-HT.

The lung is a site at which the reuptake and removal of circulating 5-HT takes place. Different components of cigarette smoke have been shown to reduce MAOs' activity in the lung [29]–[32]. An inhibition of the reuptake and removal of 5-HT may lead to the accumulation of 5-HT in the pulmonary vasculature, a process believed to be involved in the pathogenesis of lung disease [33], including COPD.

### Limitations

The present study has significant limitations. Firstly, it is a cross-sectional study and thereby does not allow for the conclusion of a causal link between 5-HT and the presence of COPD. Prospective cohort studies on healthy smokers are therefore required to fully address the role of 5-HT in the development and progression of COPD. Secondly, all measurements were carried out in plasma, which reflects only systemic changes and may not adequately reflect the local concentrations in the lungs. Further studies involving biological samples such as bronchoalveolar lavage (BAL), induced sputum and exhaled breath condensate, might shed more light on the local pulmonary processes. Thirdly, the comparison of plasma 5-HT levels between healthy non-smokers and COPD non-smokers was limited by the small sample size; furthermore, the large difference in sample size between the two groups may not provide a fair comparison. The age difference between COPD patients and control subjects is another limitation in this study. To ensure our results were not due to the age difference between the two groups, age was entered as a covariant in the regression analyses.

### Conclusion

Our results demonstrated the elevation of plasma 5-HT levels in COPD patients, which is a significant mediator of the relation between cigarette smoking and the presence of COPD. We speculate that the cigarette smoke-mediated injury in COPD lungs is partially mediated by plasma 5-HT levels. Both cigarette smoking and COPD affected the association between plasma 5-HT levels and age. Our findings further support the involvement of the serotoninergic system in the pathogenesis of COPD. Further investigation of the functional role and the regulation of 5-HT in COPD is important for understanding and preventing the progression of this disease.
